# *Inonotus hispidus* Protects against Hyperlipidemia by Inhibiting Oxidative Stress and Inflammation through Nrf2/NF-κB Signaling in High Fat Diet Fed Mice

**DOI:** 10.3390/nu14173477

**Published:** 2022-08-24

**Authors:** Yongfeng Zhang, Jie Hao, Zijian Liu, Zhige Li, Lirong Teng, Di Wang

**Affiliations:** 1 School of Life Sciences, Jilin University, Changchun 130012, China; 2 Engineering Research Center of Chinese Ministry of Education for Edible and Medicinal Fungi, Jilin Agricultural University, Changchun 130118, China; 3 Joint International Research Laboratory of Modern Agricultural Technology, Ministry of Education, Jilin Agricultural University, Changchun 130118, China

**Keywords:** *Inonotus hispidus*, hypolipidemic, oxidative stress, inflammation, Nrf2/NF-κB

## Abstract

Obesity is frequently associated with dysregulated lipid metabolism and lipotoxicity. *Inonotus hispidus* (Bull.: Fr.) P. Karst (IH) is an edible and medicinal parasitic mushroom. In this study, after a systematic analysis of its nutritional ingredients, the regulatory effects of IH on lipid metabolism were investigated in mice fed a high-fat diet (HFD). In HFD-fed mice, IH reversed the pathological state of the liver and the three types of fat and significantly decreased the levels of low-density lipoprotein cholesterol (LDL-C), total cholesterol (TC), triglycerides (TG), and leptin (LEP) and increased the level of high-density liptein cholesterol (HDL-C) in serum. Meanwhile, IH ameliorated liver damage by reducing alanine aminotransferase (ALT), aspartate aminotransferase (AST), interleukin (IL)-1β, IL-6, tumor necrosis factor-alpha (TNF-α), and plasminogen activator inhibitor-1 (PAI-1) levels in the liver and serum. Compared with HFD-fed mice, IH significantly modulated the gut microbiota, changed the relative abundances of microflora at different taxonomic levels, and regulated lipid levels. The results showed that 30 differential lipids were found. Results from Western blotting confirmed that IH regulated the nuclear factor erythroid-2 related factor 2 (Nrf2)/nuclear factor-kappa B (NF-κB) signaling pathway and oxidative stress. This study aimed to provide experimental evidence for the applicability of IH in obesity treatment.

## 1. Introduction

Obesity is a pathological condition requiring clinical intervention [1]; it may induce complications involving metabolic disorders, including type II diabetes and hyperlipidemia [2,3]. In China, from 2015 to 2019, the prevalence of obesity was 3.6%, 7.9%, and 16.4% among children under the age of 6 years, adolescents aged 6–17 years, and adults (≥18 years), respectively [4]. The increasing burden of obesity has grave implications for individuals, families, and societies.

Obesity is frequently associated with dysregulated lipid metabolism and lipotoxicity [5], which may induce increased hepatic fat uptake and new fat synthesis in the liver. However, the compensatory enhancement of fatty acid oxidation fails to normalize lipid levels, ultimately triggering oxidative stress and leading to cellular damage and the occurrence of non-alcoholic fatty liver disease [6]. Furthermore, certain gut microbes influence the occurrence and development of obesity by improving intestinal oxidative stress, inhibiting intestinal inflammation, and maintaining intestinal barrier integrity [3,7]. Obesity is associated with elevated levels of markers of oxidative stress and low-grade systemic inflammation [8]. An excessive accumulation of white adipose tissue leads to adipocyte hypertrophy, metabolic dysregulation, and high expression levels of pro-inflammatory adipokines, such as interleukin (IL)-6 and tumor necrosis factor-alpha (TNF-α) [9]. TNF-α stimulates the nuclear factor-kappa B (NF-κB) signaling pathway to regulate lipolysis and activation; it increases the phosphorylation of insulin receptor substrate 1 (IRS1), which further stimulates the overproduction of reactive oxygen species (ROS) [8]. A key transcription factor regulating the cytoprotective pathways of antioxidants, nuclear factor erythroid-2 related factor 2 (Nrf2), is downregulated in diabetes, hypertension, and inflammation [10]. Therefore, Nrf2/NF-κB-mediated lipid metabolism and gut microbiota may be targets for obesity treatment.

Currently, weight loss occurs primarily through diet- and exercise-related strategies, which are often not sustainable [11]. Bariatric surgery is an effective and drastic intervention; however, its side effects are also obvious [11]. Therefore, there is an urgent need to explore safe and effective ways to prevent and treat obesity. Mushrooms are rich in polysaccharides and proteins, low in fat, and cholesterol-free [12]. The intake of dietary fiber obtained from mushrooms may reduce the levels of total cholesterol (TC) and low-density lipoprotein (LDL) and ameliorate serum lipid levels [13]. *Grifola frondosa* modulates ceramide levels and restores lipid metabolism by inhibiting the Toll-like receptor 4/NF-κB signaling associated with inflammation and insulin resistance (IR) to treat obesity [14]. *Inonotus hispidus* (Bull.: Fr.) P. Karst (IH) is an edible and medicinal parasitic mushroom belonging to the phylum Basidiomycota, class Agaricomycetes, and family *Hymenochaetaceae*. IH is mainly distributed in the Hebei Chengde, Shandong Linqing, Xiajin, and Xinjiang Aksu areas, and it prefers to live on mulberry, water willow, elm, poplar, and Japanese acacia. Previous investigations on IH have mainly focused on chemical composition analysis, artificial cultivation, mycelium fermentation, and pharmacological activity studies [15,16,17,18,19]. IH promotes the activation of human T cells, increases natural killer cell activity, and induces dendritic cell maturation [20]. IH petroleum ether extract exhibits antitumor activity by modulating energy production as well as amino acid and steroid hormone biosynthetic pathways in H22-tumor-bearing mice [21]. Extracellular exopolysaccharides in IH can protect livers in mice with acute alcoholic liver injury by activating the Nrf2 signaling pathway and increasing the expression levels of downstream antioxidant enzymes such as catalase (CAT) and superoxide dismutase (SOD) [22]. However, the potential hypolipidemic and hypoglycemic effects of IH in mice with diet-induced obesity (DIO) and the underlying mechanisms have not been systematically investigated.

In this study, based on the detection of the main components of IH, combined with gut microbiota and lipid metabolomic analyses, the role of IH in regulating oxidative stress and inflammation, based on the Nrf2/NF-κB signaling pathway, to alleviate obesity symptoms was explored in C57BL/6 mice with DIO. Our data provide experimental evidence for the applicability of IH in obesity treatment.

## 2. Materials and Methods

### 2.1. Detection of IH Components

IH fruiting bodies from Linqing, Shandong, were identified by Professor Yu Li. The general components (total dietary fiber, ash, total sugar, fat, protein, reducing sugars, total saponin, total flavonoids, total sterol, etc.), twenty kinds of amino acids, thirty-five kinds of fatty acids, seven kinds of minerals, six kinds of heavy metals, eight kinds of vitamins, and five kinds of nucleotides in IH were systematically detected, as we previously described [14,23].

### 2.2. Animal Experiments and Agent Administration Protocol

All experiments were performed in accordance with the guidelines of the Institutional Animal Ethics Committee of Jilin University (SY202106003). Thirty-six male C57BL/6JGpt mice (5 weeks old) from GemPharmatech Co., Ltd. (Nanjing, China; SCXK [SU] 2018-0008) were maintained on either a normal chow diet (NCD; D12450B; 10% kcal fat, 20% kcal protein, and 70% kcal carbohydrate; Liaoning Changsheng Biotechnology Co., Ltd., Benxi, China) or a high-fat diet (HFD; D12492; 60% kcal fat, 20% kcal protein, and 20% kcal carbohydrate; Xiao Shu You Tai Biotechnology Co., Ltd., Beijing, China) under specific-pathogen-free (SPF) conditions on a 12 h light/dark cycle at a constant temperature (23 ± 1 °C) and humidity (40–60%) for 8 weeks. To establish the DIO model for the high-fat diet study, 24 randomly selected mice were fed an HFD ad libitum during the entire experimental period. From week 9, the DIO mice were randomly divided into four groups (*n* = 6/group), including an intragastrically HFD-fed group with 5 mL/kg of normal saline, an intragastrically simvastatin (SV) (SFDA Approval No.: H20093943, Chengdu Hengrui Pharmaceutical Co., Ltd., Chengdu, China)-treated group with 3 mg/kg of SV, and low- and high-dose intragastrically IH-treated groups with 500 and 1000 mg/kg of IH, respectively, daily for 8 weeks. The NCD mice were randomly divided into two groups (*n* = 6/group), including the vehicle-treated intragastrically NCD-fed group with 5 mL/kg of normal saline and the intragastrically IH-treated NCD-fed group with 500 mg/kg of IH daily for 8 weeks. Weekly measurements of body weight and plasma glucose levels were performed for all mice. After 8 weeks of drug treatment, the mice were euthanized using CO_2_ (the CO_2_ replacement rate was 30–70% of the container volume per minute). Peripheral blood was obtained by sampling the retro-orbital venous plexus. Organs (heart, liver, spleen, and kidney), epididymal white adipose tissue (eWAT), inguinal white adipose tissue (iWAT), and perirenal white adipose tissue (pWAT) were dissected and weighed. The above tissue parts were stored at −80 °C for further biochemical analysis, and the remaining parts were fixed in a 4% tissue fixative (BL539A, Biosharp, Guangzhou, China) for subsequent pathological analysis.

### 2.3. Cytokine Detection

Liver tissue was homogenized in normal saline, and the protein concentration was determined using a Pierce™ bicinchoninic acid (BCA) Protein Assay Kit (23225; Thermo Scientific^TM^, Waltham, MA, USA). Serum was collected twice by centrifugation at 3500 rpm at 4 °C for 10 min. The levels of alanine aminotransferase (ALT) (MM-44625M1), aspartate aminotransferase (AST) (MM-44115M1), IL-1β (MM-0040M1), IL-6 (MM-0163M1), TNF-α (MM-0132M1), and plasminogen activator inhibitor-1 (PAI-1) (MM-0066M1) in the liver and serum, ROS (MM-43700M1), malondialdehyde (MDA) (MM-0897M1), and lysophosphatidylcholine (LPC) (MM-44698M1) in the liver, and high-density liptein cholesterol (HDL-C) (MM-44105M1), low-density lipoprotein cholesterol (LDL-C) (MM-43685M1), total cholesterol (TC) (MM-0632M1), triglycerides (TG) (MM-0631M1), and leptin (LEP) (MM-0622M1) (Meimian Biotechnology, Yancheng, China) in the serum were measured using enzyme-linked immunosorbent assay (ELISA) kits.

### 2.4. Histopathological Analysis

Hematoxylin and eosin (H&E) staining and Oil Red O staining were performed as described in our previous study [14]. Fixed adipose tissue (eWAT, iWAT, and pWAT) and organ tissue (heart, liver, spleen, and kidney) were embedded in paraffin, sectioned at 5 µm, and stained with H&E. Frozen liver tissue was sectioned at 10 µm, fixed, and stained with Oil Red O and H&E. All specimens were observed and photographed using an Eclipse Ci-L upright microscope (Nikon Corporation, Tokyo, Japan).

### 2.5. Intestinal Microflora Analysis

A gut microbiota analysis of mouse cecum content in samples from NCD-, HFD-, and IH-treated groups (500 mg/kg) (*n* = 3/group) was performed by 16S rRNA sequencing using an Illumina NovaSeq platform at Shanghai Personalbio Technology Co., Ltd. (Shanghai, China), as described in our previous study [14]. Sequence sets with 97% identity in 16S rDNA gene sequences were clustered into operational taxonomic units (OTUs). The alpha diversity index included Chao1, observed species, Shannon, Faith’s phylogenetic diversity (Faith’s PD), Pielou’s evenness, and Good’s coverage, and the significance of differences was verified by the Kruskal–Wallis rank-sum test and Dunn’s test as post hoc tests. The beta diversity was calculated by the Bray–Curtis. When the linear discriminant analysis (LDA) score was >2, the results of the LDA effect size (LEfSe) analysis, performed to identify the biomarkers for each group, were more reliable.

### 2.6. Plasma Lipidome Analysis

A plasma lipidome analysis of mouse serum from the NCD-, HFD-, and IH-treated groups (500 mg/kg) (*n* = 3/group) was performed by liquid chromatography–mass spectrometry (LC–MS) at Shanghai Personalbio Technology Co., Ltd. (Shanghai, China), as described in our previous study [14]. According to the detected compounds, an orthogonal partial least squares discriminant analysis (OPLS-DA) was used for a metabolite variation analysis. Statistically significant differences (*p* ≤ 0.05) in metabolite levels and variable importance in projection (VIP) values ≥ 1.0 were regarded as the standard for differential lipids to filter out biomarkers.

### 2.7. Western Blotting

Total proteins were extracted from the collected liver tissues using a radioimmunoprecipitation assay (RIPA) buffer (PC101, EpiZyme, Shanghai, China) containing protease and phosphatase inhibitors (P002, New Cell & Molecular Biotech Co., Ltd., Suzhou, China) and were homogenized using a high-throughput tissue grinder (SCIENTZ-48, Ningbo Scientz Biotechnology Co., Ltd., Ningbo, China). After denaturation, 40 μg of protein was separated by 10% sodium dodecyl sulfate-polyacrylamide gel electrophoresis (SDS-PAGE) (PG112, Shanghai Epizyme Biomedical Technology Co., Ltd., Shanghai, China) and then transferred onto a polyvinylidene fluoride (PVDF) membrane (10600023, Cytiva, Marlborough, MA, USA). Membranes were blocked by Rapid Closure solution (P30500, New Cell & Molecular Biotech Co., Ltd., Suzhou, China) and incubated with the primary antibodies overnight and then the secondary antibodies for 4 h at 4 °C. Finally, immunoreactive bands were visualized using an automated chemiluminescence image analysis system (Tanon 5200, Tanon Science & Technology Co., Ltd., Shanghai, China) and Ultra High Sensitivity enhanced chemiluminescence (ECL) kits (GK10008, GLPBIO, Montclair, NJ, USA). Protein expression levels were measured using ImageJ software (National Institutes of Health, Bethesda, MD, USA) and normalized to glyceraldehyde-3-phosphate dehydrogenase (GAPDH). Details regarding the antibodies used in this work are presented in Table S1.

### 2.8. Statistical Analysis

All values are presented as means ± SD. Biochemical indices were compared between different groups by one-way analysis of variance (ANOVA) followed by Tukey’s test using BONC DSS Statistics 25 (IBM, Armonk, NY, USA). Differences were considered statistically significant at *p* < 0.05.

## 3. Results

### 3.1. Main Composition of IH

The general nutritional composition of IH is 45.90% total dietary fiber, 25.50% total sugar, 15.90% protein, 9.90% ash, 9.22% total flavonoids, 6.18% moisture, 5.04% total polyphenols, 4.70% fat, 1.48% total triterpenes, 0.50% total saponins, 0.46% total alkaloids, 0.31% total sterols, etc. Among them, the total dietary fiber content was the highest. The glutamic acid content was the highest among the 20 amino acids detected. Seven minerals, including calcium (Ca), iron (Fe), zinc (Zn), selenium (Se), potassium (K), sodium (Na), and manganese (Mn), were detected, in addition to low concentrations of six heavy metals (lead (Pb), arsenic (As), mercury (Hg), cadmium (Cd), copper (Cu), and chromium (Cr)). Eight vitamins and five nucleotides were also detected (Table 1). The correlation spectra were presented in Figure S1.

### 3.2. Hypolipidemic Effects of IH in HFD-Fed Mice

**Figure 1:**
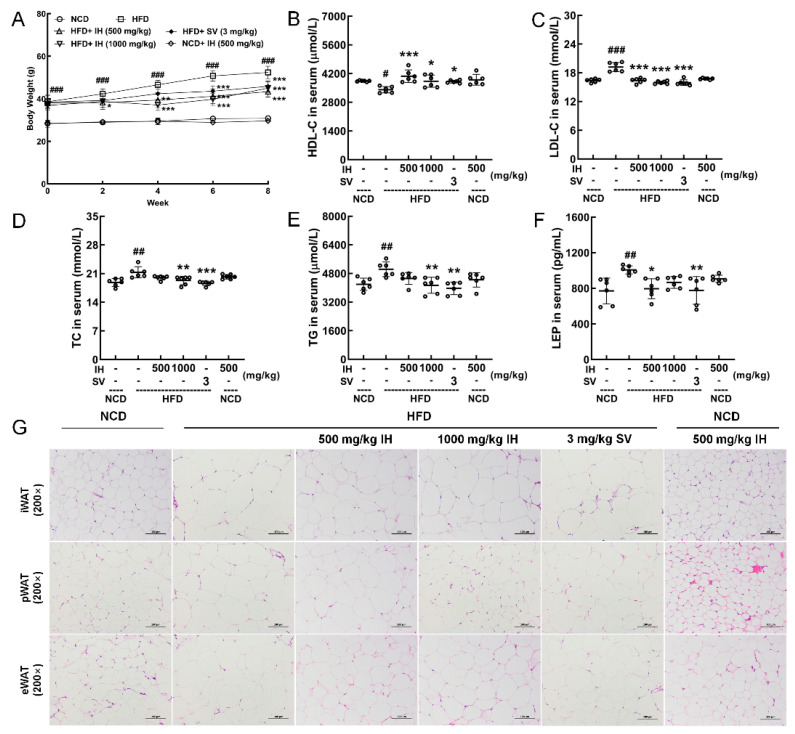
IH suppressed HFD-induced obesity and hyperlipidemia. (**A**) IH inhibited body weight gain in HFD mice (*n* = 6). IH administration increased the level of (**B**) HDL-C and decreased the levels of (**C**) LDL-C, (**D**) TC, (**E**) TG, and (**F**) LEP in the serum of HFD-fed mice. (**G**) H&E staining of iWAT, pWAT, and eWAT (200×; scale bar: 100 μm). Data were analyzed using a one-way ANOVA and expressed as the means ± SD. ^#^ *p* < 0.05, ^##^ *p* < 0.01, and ^###^ *p* < 0.001 versus vehicle-treated NCD-fed mice; * *p* < 0.05, ** *p* < 0.01, and *** *p* < 0.001 versus vehicle-treated HFD-fed mice.

IH and SV gradually and significantly inhibited weight gain in HFD-fed mice (*p* < 0.05) (Figure 1A). Compared with vehicle-treated HFD-fed mice, IH, especially at 1000 mg/kg, strongly enhanced the levels of HDL-C (*p* < 0.05) (Figure 1B) and suppressed the levels of LDL-C (*p* < 0.001) (Figure 1C), TC (*p* < 0.01) (Figure 1D), and TG (*p* < 0.01) (Figure 1E) in serum. At 500 mg/kg, IH lowered the level of LEP (*p* < 0.05) (Figure 1F). H&E staining revealed a significantly increased volume of adipocytes in vehicle-treated HFD-fed mice, which was strongly suppressed by IH and SV (Figure 1G). Among the experimental groups, no significant changes were noted in organ (heart, spleen, and kidney) structures (Figure S2). Compared with vehicle-treated HFD-fed mice, the plasma glucose levels of mice exhibited a reduction after IH administration, especially in week 6 (*p* < 0.05) (Table 2). IH significantly reduced liver indices (500 mg/kg, *p* < 0.001) without affecting other organs (Table 2). IH alone showed no significant effects on the above-mentioned factors in NCD-treated mice (Figure 1, Figure S2 and Table 2).

### 3.3. IH Ameliorated Hepatic Steatosis in HFD-Fed Mice

**Figure 2:**
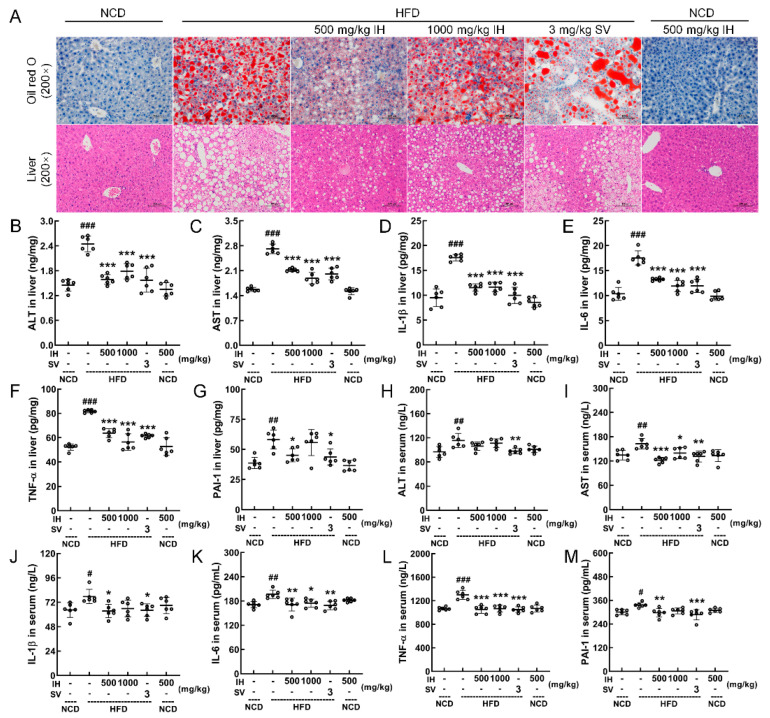
IH attenuated damage and inflammation in the liver. (**A**) Histopathological analysis of the liver by Oil Red O staining (200×; scale bar: 100 μm) and H&E staining (200×; scale bar: 100 μm). IH suppressed the liver levels of (**B**) ALT, (**C**) AST, (**D**) IL-1β, (**E**) IL-6, (**F**) TNF-α, and (**G**) PAI-1 and serum levels of (**H**) ALT, (**I**) AST, (**J**) IL-1β, (**K**) IL-6, (**L**) TNF-α, and (**M**) PAI-1 in the HFD-fed mice but had no significant effect on (**H**) ALT. Data were analyzed using a one-way ANOVA and expressed as means ± SD (*n* = 6). ^#^ *p* < 0.05, ^##^ *p* < 0.01, and ^###^ *p* < 0.001 versus vehicle-treated NCD-fed mice; * *p* < 0.05, ** *p* < 0.01, and *** *p* < 0.001 versus vehicle-treated HFD-fed mice.

In HFD-fed mice, a large amount of lipid accumulation (Oil Red O staining), high degrees of lipid deposition, and large amounts of lipid droplets (H&E staining) were improved by IH and SV (Figure 2A). Moreover, in IH- and SV-treated HFD-fed mice, low levels of ALT (*p* < 0.001) (Figure 2B), AST (*p* < 0.001) (Figure 2C), IL-1β (*p* < 0.001) (Figure 2D), IL-6 (*p* < 0.001) (Figure 2E), and TNF-α (*p* < 0.001) (Figure 2F) in the liver were observed. At 500 mg/kg, IH decreased the hepatic levels of PAI-1 (*p* < 0.05) (Figure 2G) in HFD-fed mice, and SV was also valid (*p* < 0.05). In the serum of HFD-fed mice, IH and SV showed the same effects on AST (*p* < 0.05) (Figure 2I), IL-6 (*p* < 0.05) (Figure 2K), TNF-α (*p* < 0.001) (Figure 2L), and PAI-1 (*p* < 0.01) (Figure 2M). At 500 mg/kg, IH decreased the serum levels of IL-1β (*p* < 0.05) (Figure 2J) and PAI-1 (*p* < 0.01) (Figure 2M) in HFD-fed mice, and SV was also valid (*p* < 0.05). IH failed to influence the serum levels of ALT (Figure 2H). IH alone had no significant effect on the levels of these enzymes and cytokines in NCD-treated mice (Figure 2B–M).

### 3.4. IH Regulated Intestinal Microflora in HFD-Fed Mice

**Figure 3:**
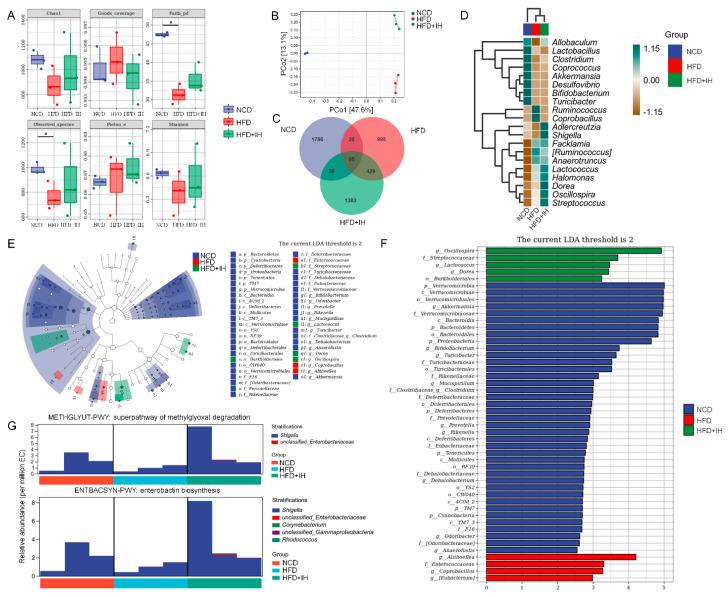
IH regulated the intestinal microflora. (**A**) Chao1, Shannon, Pielou’s evenness, observed species, Faith’s PD, and Good’s coverage index values from alpha diversity analysis among vehicle-treated NCD-fed mice, vehicle-treated HFD-fed mice, and IH-treated HFD-fed mice (*n* = 3). ^#^ *p* < 0.05 versus vehicle-treated NCD-fed mice. (**B**) PCoA of unweighted UniFrac distance from beta diversity analysis. (**C**) Venn diagram. (**D**) Heatmap of the top 20 genera by average abundance, using UPGMA clustering according to the Euclidean distance of species composition. (**E**) Taxonomic cladogram and (**F**) histogram of distribution of LDA values of significantly different species according to LefSe analysis. (**G**) Species composition in significantly different metabolic pathways via metagenomeSeq.

Imbalances involving intestinal microflora are related to abnormal lipid metabolism and are involved in the pathogenesis of obesity [24]. In intestinal microflora analysis, alpha diversity was used to reflect the richness, diversity, and uniformity of species within any sample [25]. Between the vehicle- and IH-treated HFD-fed mice, IH showed no effects on alpha diversity (Figure 3A). Beta diversity was used to represent differences in species composition. IH administration resulted in a distinct separation of the intestinal microflora composition in mice (PCo2, 13.1%, Figure 3B). A Venn diagram was used to represent the intestinal microbial community, and the number of OTUs in each collection was determined based on OTU abundance. Among the 4763 OTUs detected in the experimental groups, 95 were common among all. The number of specific OTUs was 1786 (37.50%) in vehicle-treated NCD-fed mice, 995 (20.89%) in vehicle-treated HFD-fed mice, and 1383 (29.04%) in IH-treated HFD-fed mice, indicating a strong influence of IH on the composition of intestinal flora (Figure 3C). A heatmap analysis of the flora with the top 20 average abundances at the genus level showed that IH treatment increased the average abundance of *Allobaculum*, *Adlercreutzia*, *Shigella*, *Dorea*, *Oscillospira*, and *Streptococcus* and decreased *Ruminococcus* and *Coprobacillus* compared with the vehicle-treated HFD-fed mice (Figure 3D). An LEfSe analysis was performed to identify species with significantly different abundance across experimental groups and identify stable and differential landmark species at all taxonomic levels [26]. IH significantly increased the relative abundances of *Burkholderiales*, *Streptococcaceae*, *Lactococcus*, *Dorea*, and *Oscillospira* in HFD-fed mice (Figure 3E). *Oscillospira* had the highest LDA value (Figure 3F). Between the vehicle- and IH-treated HFD-fed mice, 18 significantly altered taxa were noted (Table S2). Differential metabolic pathways were detected by metagenomeSeq (Table S3), and the superpathways of methylglyoxal (MGO) degradation and enterobactin biosynthesis were significantly upregulated by IH in HFD-fed mice (Figure 3G) and were related to the abundance of *Shigella*.

### 3.5. IH Regulated Lipid Metabolism

**Figure 4:**
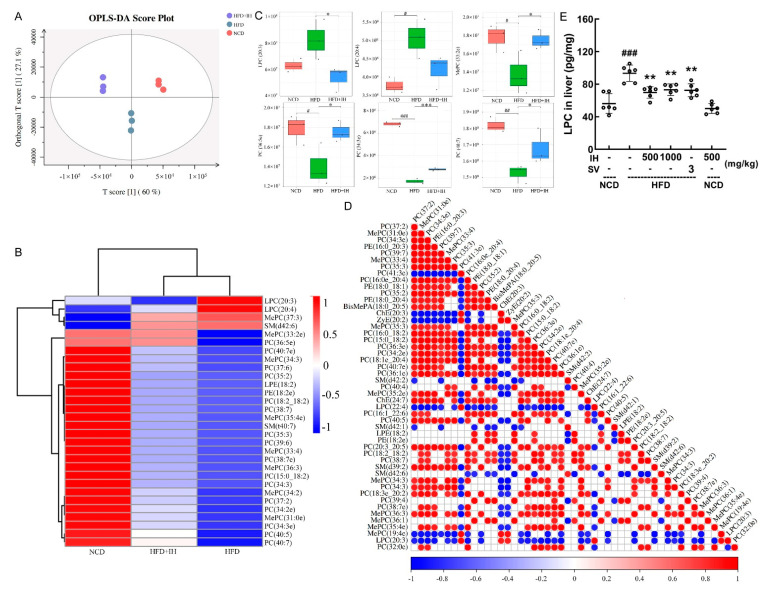
IH regulated lipid metabolites in HFD-fed mice. (**A**) OPLS-DA score plot. (**B**) Heatmap of significantly altered metabolites. (**C**) Boxplots of representative differential lipids affected by IH (*n* = 3). (**D**) The associated heatmap of associated lipids. (**E**) IH decreased the level of LPC in the livers of HFD-fed mice. ^#^ *p* < 0.05, ^##^ *p* < 0.01, and ^###^ *p* < 0.001 versus vehicle-treated NCD-fed mice; * *p* < 0.05, ** *p* < 0.01 and *** *p* < 0.001 versus vehicle-treated HFD-fed mice.

Lipidomic analysis can identify and quantify lipids in serum, aiding the study of lipid metabolism mechanisms [27]. OPLS-DA was used to analyze differences between groups (Figure 4A). VIP ≥ 1 and *p* ≤ 0.05 were used as the criteria for screening differential lipids. IH increased the levels of 26 lipids and decreased the levels of 4 lipids (Table S4). These differential lipids were analyzed by hierarchical clustering and are expressed visually (Figure 4B). LPC (20:3), LPC (20:4), phosphatidylcholine (PC) (36:5e), and MePC (33:2e) were the most pronounced lipids that were reversed by IH (Figure 4B). The levels of LPC (20:3), LPC (20:4), MePC (33:2e), PC (36:5e), PC (34:3e), and PC (40:7) are shown in Figure 4C. LPC was significantly negatively correlated with all PCs except PC (41:3e) (*p* < 0.05) (Figure 4D). IH and SV significantly reversed the hepatic level of LPC in HFD-fed mice (*p* < 0.01) (Figure 4E).

### 3.6. IH Regulated Nrf2/NF-κB Pathway and Oxidative Stress

**Figure 5:**
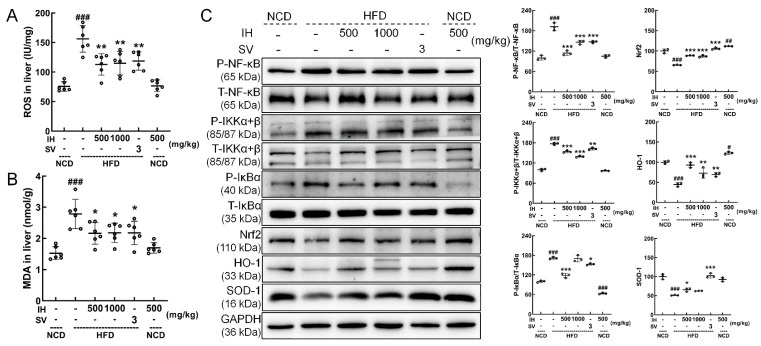
IH had significant antioxidant activities. Compared with vehicle-treated HFD-fed mice, IH decreased the levels of (**A**) ROS and (**B**) MDA in the liver. (**C**) IH influenced the Nrf2/NF-κB pathway in the livers of HFD-fed mice. IH administration decreased the phosphorylation levels of NF-κB, IKKα + β, and IκBα and increased the levels of Nrf2, HO-1, and SOD-1 in the livers of HFD-fed mice. Quantification data were normalized to GAPDH or the corresponding total protein concentration and expressed as the percentage of vehicle-treated NCD-fed mice. Data were analyzed using a one-way ANOVA and expressed as the means ± SD (*n* = 3). ^#^ *p* < 0.05, ^##^ *p* < 0.01, and ^###^ *p* < 0.001 versus vehicle-treated NCD-fed mice; * *p* < 0.05, ** *p* < 0.01, and *** *p* < 0.001 versus vehicle-treated HFD-fed mice.

The hepatic levels of ROS (*p* < 0.01) (Figure 5A) and MDA (*p* < 0.05) (Figure 5B) were decreased in IH- and SV-treated HFD-fed mice. Compared with vehicle-treated HFD-fed mice, IH significantly downregulated the phosphorylation levels of the inflammation-related factors NF-κB (*p* < 0.001), IKKα + β (*p* < 0.001), and IκBα (500 mg/kg, *p* < 0.001) and significantly upregulated the expression levels of Nrf2 (*p* < 0.001), HO-1 (*p* < 0.01), and SOD-1 (500 mg/kg, *p* < 0.05). Compared with the vehicle-treated NCD-fed mice, IH alone only increased the levels of Nrf2 (*p* < 0.01) and HO-1 (*p* < 0.05) and suppressed the activation of P-IκBα (*p* < 0.001) (Figure 5C).

## 4. Discussion

IH is rich in dietary fiber, which helps to improve hyperlipidemia by affecting lipid metabolism [28] and has a positive effect on weight loss [29], suggesting a material basis for its hypolipidemic effect. IH significantly improved the pathological state and function of adipocytes. Changes in the levels of TC, TG, LDL-C, and HDL-C (related to hyperlipidemia [30]) revealed the anti-hyperlipidemic effect of IH in HFD-fed mice. LEP is an adipokine secreted by adipocytes that reflects the degree of obesity [31]; a decline in the level of LEP revealed the anti-obesity effect of IH in HFD-fed mice. IH significantly suppressed the levels of AST, ALT, and PAI-1 and inhibited hepatic steatosis in HFD-fed mice, confirming its hepatoprotective effect, which is the center of lipid metabolism [32]. The reduction in the levels of ROS and MDA demonstrated the antioxidant activity of IH in HFD-fed mice.

In obese mice, IH increased the abundance of the genera *Allobaculum*, *Dorea*, and *Oscillospira*, facilitating the production of short-chain fatty acids (SCFAs) related to metabolic processes [33,34,35] while decreasing the abundance of the genera *Ruminococcus* and *Coprobacillus*. SCFAs such as acetate, propionic acid, and butyric acid can reduce the generation of pro-inflammatory cytokines [36], displaying resistance to intestinal inflammation [37]. SCFAs can suppress appetite, promote lipid oxidation rather than lipid production, and reduce the storage of white adipose tissue [38,39]. Dietary fiber can mitigate the reduction in SCFAs caused by a high-fat diet [40], which has been consistent with our present data. Accordingly, as a beneficial bacteria genus [41], *Allobaculum* can regulate hepatic lipid metabolic processes [42], shows a negative correlation with obesity [43], and its supplementation can reduce the rapid weight gain caused by a high-fat diet [44]. *Allobaculum* plays a role in suppressing inflammatory responses by reducing the expression of p-IKK and TNF-α [45], which has been confirmed in our results. As a healthy bacteria genus [41], *Dorea* is negatively associated with inflammatory diseases [46]. Both *Oscillospira* and *Ruminococcus* are inflammatory bacteria associated with inflammatory bowel disease [47,48], and *Oscillospira* helps to maintain lipid homeostasis [49]. *Rosa Roxburghii Tratt*, possessing hypolipidemic effects, can reduce the abundance of *Coprobacillus* [50]. Moreover, *Streptococcus* may be the main force for decomposing a large amount of cellulose [51], which increased in IH-treated HFD-fed mice. Based on the changes induced by IH on the abundance of intestinal microbes, IH might upregulate the superpathway of MGO degradation and enterobactin biosynthesis. The impairment of enterobactin biosynthesis elevates ROS levels [52]. Meanwhile, MGO can activate the oxidative pathway and induce inflammation [53,54], and the formation and accumulation of MGO are strongly associated with obesity [55].

Metabolites of intestinal flora affect the process of lipid metabolism and host lipid composition [56]. IH could significantly decrease the level of LPC in HFD-fed mice. As a biologically active pro-inflammatory lipid molecule, LPC promotes the expression of genes involved in cholesterol biosynthesis, thereby participating in lipid metabolic processes [57]. LPC is formed by the hydrolysis of PC in LDL and cell membranes via phospholipase A (2) or oxidation [58], and impairment in PC biosynthesis is associated with the occurrence of fatty liver disease [59]. PC has excellent antioxidant properties, can scavenge ROS, prevents lipid peroxidation, and is known as an antioxidant/anti-inflammatory phospholipid [60]. Additionally, changes in the MGO level are, in turn, related to changes in the metabolic processes involving PC [61]. MGO increases oxidative stress levels, which can lead to lipid peroxidation through the production of ROS and ultimately lead to cell damage [54,61]. IH regulated lipid metabolism and showed anti-hyperlipidemic activity related to its modulation of intestinal flora, which further influenced the levels of metabolites.

Oxidative stress leads to increased lipid peroxidation and is closely associated with hyperlipidemia-related tissue damage [62,63]. Nrf2, a key regulator of antioxidant responses and maintaining cellular redox hemostasis, can inhibit lipogenesis [64] and nullify ROS [65] by promoting the expression of a series of downstream antioxidant genes, such as *SOD* and *HO-1* [66]. SOD-1 is one of the three distinct isoforms identified of SOD in mammals. SOD isoforms scavenge superoxide radicals and reduce their toxicity [67]. *HO-1*, a key target gene of Nrf2, exerts antioxidant effects by resisting endogenous and exogenous stimuli [68]. Nrf2/HO-1 signaling contributes to the scavenging of lipid peroxides [69].

Oxidative stress and inflammation are inextricably associated [70]. Nrf2 can negatively regulate NF-κB signaling during cellular inflammatory responses [10]. Under typical physiological conditions, NF-κB is in a normal binding state with IκBα [71], whereas cellular injury caused by various factors such as pro-inflammatory factors or ROS triggers the activation of the IKK complex, which leads to IκBα phosphorylation, ubiquitination by the ubiquitin ligase system (ULS), and the release of NF-κB dimers. These NF-κB dimers enter the nuclear membrane, thereby initiating the transcription of target genes [72,73]. Activated NF-κB in hepatocytes promotes liver inflammation [74] and induces the transcription of inflammatory cytokine genes such as TNF-α and IL-6 [75]. TNF-α can promote lipolysis and increase the release of free fatty acids, thereby promoting adipogenesis [76]. According to the present data, IH could regulate lipid metabolic processes through the Nrf2/NF-κB pathway and had a relevant effect on oxidative stress as well as the inflammatory response in this process.

The present study had certain limitations. In this study, we first reported the hypolipidemic effects of IH and analyzed the components involved. However, the specific active components possessing hypolipidemic activity were not confirmed; thus, further investigation is needed.

## 5. Conclusions

In conclusion, IH regulated lipid metabolism through the Nrf2/NF-κB signaling pathway, which is closely related to oxidative stress and inflammatory responses, in HFD-fed mice. Our study provides insights into the application of IH as a hypolipidemic agent and will facilitate its commercial application.

## Figures and Tables

**Table 1:** The main composition of IH.

	**Compounds**	**Contents (%)**	**Compounds**	**Contents (%)**
General nutritional composition	Total dietary fiber	45.90	Total triterpenes	1.48
	Total sugar	25.50	Reducing sugar	1.29
	Protein	15.90	Soluble dietary fiber	0.68
	Ash	9.90	Soluble sugar	0.57
	Total flavonoids	9.22	Total saponins	0.50
	Moisture	6.18	Total alkaloids	0.46
	Total polyphenols	5.04	Total sterols	0.31
	Fructose	4.81	Glucose	UD*^a^*
	Mannitol	4.81	Lactose	UD*^a^*
	Fat	4.70	Maltose	UD*^a^*
	Soluble protein	2.20	Sucrose	UD*^a^*
Amino acids	Glutamic acid	1.83	Isoleucine	0.51
	Aspartic acid	1.15	Phenylalanine	0.50
	Leucine	0.85	Proline	0.45
	Lysine	0.68	Tryptophan	0.29
	Alanine	0.65	Tyrosine	0.27
	Valine	0.61	Histidine	0.23
	Arginine	0.60	Glutamine	0.21
	Threonine	0.60	Methionine	0.09
	Glycine	0.59	Asparagine	0.07
	Serine	0.58	Cysteine	0.02
Fatty acids	C18:2n6c	1.074	C18:3n3	UD*^b^*
	C18:1n9c	0.479	C20:1	UD*^b^*
	C16:0	0.291	C20:2	UD*^b^*
	C18:0	0.060	C20:3n3	UD*^b^*
	C22:1n9	0.025	C20:3n6	UD*^b^*
	C16:1	0.013	C20:4n6	UD*^b^*
	C17:0	0.010	C20:5n3	UD*^b^*
	C15:0	0.008	C21:0	UD*^b^*
	C24:1	0.004	C22:2	UD*^b^*
	C8:0	UD*^b^*	C22:6n3	UD*^b^*
	C11:0	UD*^b^*	C23:0	UD*^b^*
	C13:0	UD*^b^*	C10:0	UD*^c^*
	C14:0	UD*^b^*	C12:0	UD*^c^*
	C14:1	UD*^b^*	C18:3n6	UD*^c^*
	C15:1	UD*^b^*	C20:0	UD*^c^*
	C17:1	UD*^b^*	C22:0	UD*^c^*
	C18:1n9t	UD*^b^*	C24:0	UD*^c^*
	C18:2n6t	UD*^b^*		
	**Compounds**	**Contents (mg/kg)**	**Compounds**	**Contents (mg/kg)**
Minerals	K	4.1 × 10^3^	Zn	93.7
	Ca	406.0	Mn	24.0
	Fe	310.0	Se	0.2
	Na	243.0		
Heavy metals	Cu	31.000	As	0.249
	Cr	30.900	Pb	0.188
	Cd	0.323	Hg	0.009
Vitamins	Vitamin B6	5.10	Vitamin A	UD*^d^*
	Vitamin D2	3.07	Vitamin B1	UD*^e^*
	Vitamin E	2.02	Vitamin C	UD*^f^*
	Vitamin B2	1.01	Vitamin D3	UD*^g^*
Nucleotides	Urine purine nucleotides	1092.13	Cytosine nucleotides	UD*^h^*
	Adenosine nucleotides	146.92	Inosinic acid	UD*^h^*
	Guanine nucleotides	44.52		

UD: undetected. UD*^a^*: the detection limit was 0.2 g/kg. UD*^b^*: the detection limit was 0.033 g/kg. UD*^c^*: the detection limit was 0.066 g/kg. UD*^d^*: the detection limit was 10 μg/kg. UD*^e^*: the detection limit was 0.06 mg/kg. UD*^f^*: the detection limit was 0.05 mg/kg. UD*^g^*: the detection limit was 7 μg/kg. UD*^h^*: the detection limit was 5 mg/kg.

**Table 2:** The effects of IH on plasma glucose and organ indices.

	Week	NCD	HFD	HFD + 500 mg/kg IH	HFD + 1000 mg/kg IH	HFD + 3 mg/kg SV	NCD + 500 mg/kg IH
Plasma glucose (mmol/L)	0	8.3 ± 1.6	9.8 ± 1.5	10.4 ± 1.9	10.9 ± 1.9	10.5 ± 2.1	8.7 ± 0.4
	2	8.9 ± 1.3	11.9 ± 1.8 ^##^	10.3 ± 1.6	10.4 ± 0.8	10.5 ± 0.7	9.2 ± 1.1
	4	8.8 ± 1.6	11.5 ± 1.8 ^#^	9.4 ± 1.4	9.8 ± 0.9	11.3 ± 1.5	9.0 ± 0.7
	6	8.3 ± 1.1	12.1 ± 2.1 ^###^	9.4 ± 0.6 *	8.7 ± 1.1 **	12.1 ± 1.5	8.6 ± 1.0
	8	8.8 ± 0.8	12.5 ± 1.1 ^##^	11.6 ± 2.7	11.6 ± 1.4	12.1 ± 1.3	8.6 ± 0.4
Organ indices (%)	Heart	0.560 ± 0.037	0.333 ± 0.055 ^###^	0.367 ± 0.044	0.353 ± 0.028	0.313 ± 0.057	0.543 ± 0.053
	Liver	3.465 ± 0.077	4.859 ± 0.576 ^###^	3.310 ± 0.334 ***	4.020 ± 0.551	4.225 ± 0.745	3.922 ± 0.281
	Spleen	0.259 ± 0.006	0.223 ± 0.029	0.225 ± 0.038	0.219 ± 0.031	0.186 ± 0.012	0.270 ± 0.011
	Kidney	1.198 ± 0.073	0.717 ± 0.044 ^###^	0.800 ± 0.142	0.801 ± 0.052	0.718 ± 0.043	1.199 ± 0.064
	Pancreas	0.641 ± 0.064	0.375 ± 0.048 ^###^	0.518 ± 0.127	0.437 ± 0.113	0.317 ± 0.048	0.662 ± 0.067

The data are expressed as means ± SD (*n* = 6). ^#^ *p* < 0.05, ^##^ *p* < 0.01, and ^###^ *p* < 0.001 versus vehicle-treated NCD-fed mice; * *p* < 0.05, ** *p* < 0.01, and *** *p* < 0.001 versus vehicle-treated HFD-fed mice.

## Data Availability

Not applicable.

## Supplementary Materials

The following supporting information can be downloaded at: https://www.mdpi.com/article/10.3390/nu14173477/s1, Figure S1: HPLC chromatograms of IH components; Figure S2: H&E staining of heart, spleen, and kidney of mice; Table S1: Details of antibodies used in Western blotting; Table S2: The taxa with significant differences between vehicle-treated HFD-fed mice and IH-treated HFD-fed mice; Table S3: The differential metabolic pathways between vehicle-treated HFD-fed mice and IH-treated HFD-fed mice; Table S4: Differential lipids between vehicle-treated HFD-fed mice and IH-treated HFD-fed mice.
