# Supplementary material for: Inonotus hispidus Protects against Hyperlipidemia by Inhibiting Oxidative Stress and Inflammation through Nrf2/NF-κB Signaling in High Fat Diet Fed Mice
Source: Nutrients. 2022 Aug 24;14(17):3477. doi: 10.3390/nu14173477 (PMC9460493; doi:10.3390/nu14173477)
Supplement: Supplementary file 1 [file nutrients-14-03477-s001.zip › nutrients-1859248-supplementary.pdf]

Table S1. Details of antibodies used in Western blotting.

Antibody	Molecular weight	Catalog number	Dilution	Company	Area
P-NF-kB p65	65 kDa	AF2006	1：1000	Affinity	Jiangsu, China
T-NF-kB p65	65 kDa	A18210	1：2000	Abclonal	Wuhan, China USA
P-IKKα+β	85/87 kDa	AF3013	1：2000	Affinity	Jiangsu, China
T-IKKα+β	85/87 kDa	bs-7557R	1：2000	Bioss	Beijing, China
P-IκBα	40 kDa	2859S	1：1000	Cell Signaling Technology	Massachusetts, USA
T-IκBα	35 kDa	A19714	1：2000	Abclonal	Wuhan, China
Nrf2	110 kDa	A1244	1：2000	Abclonal	Wuhan, China
HO-1	33 kDa	A19062	1：2000	Abclonal	Wuhan, China
SOD-1	16 kDa	A12537	1：2000	Abclonal	Wuhan, China
GAPDH	36 kDa	E-AB-20032	1：2000	Elabscience	Houston, TX, USA
Goat Anti-Rabbit (H+L)	/	E-AB-1003	1：2000	Elabscience	Houston, TX, USA
Goat-Anti-Mouse (H+L)	/	E-AB-1001	1：2000	Elabscience	Houston, TX, USA

Table S2 The taxa with significant between vehicle-treated HFD-fed mice and IH-treated HFD-fed mice.

Taxa	Abundance	LDA score	*p* value
Down-regulated flora after IH administration (Number: 9)			
Bacteria.Firmicutes.Erysipelotrichi.Erysipelotrichales.Erysipelotrichaceae._Eubacterium_._Eubacterium__dolichum	2.953	2.783	0.028
Bacteria.Firmicutes.Erysipelotrichi.Erysipelotrichales.Erysipelotrichaceae._Eubacterium_	2.953	2.760	0.028
Bacteria.Firmicutes.Erysipelotrichi.Erysipelotrichales.Erysipelotrichaceae.Coprobacillus	3.580	3.297	0.028
Bacteria.Firmicutes.Clostridia.Clostridiales.Lachnospiraceae.Blautia.Blautia_producta	2.788	2.555	0.032
Bacteria.Firmicutes.Clostridia.Clostridiales.Lachnospiraceae.Blautia	2.788	2.573	0.032
Bacteria.Bacteroidetes	3.728	3.404	0.034
Bacteria.Bacteroidetes.Bacteroidia	3.727	3.376	0.034
Bacteria.Firmicutes.Bacilli.Lactobacillales.Lactobacillaceae.Lactobacillus.Lactobacillus_vaginalis	3.209	2.820	0.034
Bacteria.Bacteroidetes.Bacteroidia.Bacteroidales	3.727	3.423	0.034
Up-regulated flora after IH administration (Number: 9)			
Bacteria.Firmicutes.Clostridia.Clostridiales.Dehalobacteriaceae	2.684	2.758	0.019
Bacteria.Firmicutes.Clostridia.Clostridiales.Dehalobacteriaceae.Dehalobacterium	2.684	2.725	0.019
Bacteria.Firmicutes.Clostridia.Clostridiales.Clostridiaceae.Clostridium.Clostridium_celatum	2.424	2.753	0.019
Bacteria.Proteobacteria.Betaproteobacteria.Burkholderiales	2.434	2.582	0.028
Bacteria.Proteobacteria.Gammaproteobacteria.Enterobacteriales	3.393	3.004	0.034
Bacteria.Proteobacteria.Gammaproteobacteria.Enterobacteriales.Enterobacteriaceae	3.393	2.976	0.034
Bacteria.Firmicutes.Clostridia.Clostridiales.Lachnospiraceae.Dorea	3.710	3.276	0.034
Bacteria.Firmicutes.Clostridia.Clostridiales.Christensenellaceae	3.917	3.431	0.034
Bacteria.Proteobacteria.Gammaproteobacteria.Enterobacteriales.Enterobacteriaceae.Shigella	3.377	2.969	0.034

The current LDA threshold is 2.

Differences were considered statistically significant at $p < 0.05$.

Table S3 The differential metabolic pathways between vehicle-treated HFD-fed mice and IH-treated HFD-fed mice.

Pathway	Description	logFC	SE	*p* values	Adj *p* values
Down-regulated pathways after IH administration (Number: 8)					
PWY-6891	thiazole biosynthesis II (Bacillus)	-1.549	0.603	0.010	0.231
METH-ACETATE-PWY	methanogenesis from acetate	-1.414	0.459	0.002	0.061
THISYN-PWY	superpathway of thiamin diphosphate biosynthesis I	-1.359	0.619	0.028	0.381
PWY-4361	S-methyl-5-thio-α-D-ribose 1-phosphate degradation	-1.050	0.522	0.044	0.448
PWY-7527	L-methionine salvage cycle III	-1.048	0.522	0.045	0.448
SO4ASSIM-PWY	sulfate reduction I (assimilatory)	-0.664	0.321	0.038	0.434
SULFATE-CYS-PWY	superpathway of sulfate assimilation and cysteine biosynthesis	-0.607	0.310	0.050	0.448
PWY-6572	chondroitin sulfate degradation I (bacterial)	-0.270	0.119	0.023	0.369
Up-regulated pathways after IH administration (Number: 33)					
PWY-6397	mycolyl-arabinogalactan-peptidoglycan complex biosynthesis	0.072	0.035	0.041	0.439
PWY-7391	isoprene biosynthesis II (engineered)	0.169	0.075	0.024	0.369
PWY-6182	superpathway of salicylate degradation	0.277	0.123	0.024	0.369
P261-PWY	coenzyme M biosynthesis I	0.457	0.072	2.372×10^{-10}	2.277×10^{-8}
PWY-7373	superpathway of demethylmenaquinol-6 biosynthesis II	0.520	0.240	0.030	0.389
UBISYN-PWY	superpathway of ubiquinol-8 biosynthesis (prokaryotic)	0.541	0.252	0.032	0.391
PWY-5178	toluene degradation IV (aerobic) (via catechol)	0.554	0.078	9.606×10^{-13}	1.230×10^{-10}
P341-PWY	glycolysis V (Pyrococcus)	0.592	0.241	0.014	0.304
PWY-6507	4-deoxy-L-threo-hex-4-enopyranuronate degradation	0.595	0.280	0.034	0.405
ALL-CHORISMATE-PWY	superpathway of chorismate metabolism	0.623	0.278	0.025	0.369
3-	4-hydroxyphenylacetate degradation	0.663	0.074	0.000	0.000

HYDROXYPHENYLACETATE-DEGRADATION-PWY					
PWY-5941	glycogen degradation II (eukaryotic)	0.689	0.159	1.391×10^{-5}	0.001
PWY-1622	formaldehyde assimilation I (serine pathway)	0.697	0.085	2.220×10^{-16}	4.263×10^{-14}
PWY-5507	adenosylcobalamin biosynthesis I (early cobalt insertion)	0.778	0.239	0.001	0.036
GLYCOL-GLYOXDEG-PWY	superpathway of glycol metabolism and degradation	0.784	0.395	0.047	0.448
LPSSYN-PWY	superpathway of lipopolysaccharide biosynthesis	0.795	0.402	0.048	0.448
PWY-6478	GDP-D-glycero-α-D-manno-heptose biosynthesis	0.802	0.269	0.003	0.078
PWY0-41	allantoin degradation IV (anaerobic)	0.880	0.316	0.005	0.138
PWY-5855	ubiquinol-7 biosynthesis (prokaryotic)	0.914	0.254	0.000	0.011
PWY-5856	ubiquinol-9 biosynthesis (prokaryotic)	0.914	0.254	0.000	0.011
PWY-5857	ubiquinol-10 biosynthesis (prokaryotic)	0.914	0.254	0.000	0.011
PWY-6708	ubiquinol-8 biosynthesis (prokaryotic)	0.914	0.254	0.000	0.011
METHGLYUT-PWY	superpathway of methylglyoxal degradation	1.010	0.483	0.036	0.423
ECASYN-PWY	enterobacterial common antigen biosynthesis	1.118	0.505	0.027	0.381
ENTBACSYN-PWY	enterobactin biosynthesis	1.148	0.503	0.022	0.369
KETOGLUCONMET-PWY	ketogluconate metabolism	1.157	0.496	0.020	0.369
PWY0-1338	polymyxin resistance	1.170	0.520	0.025	0.369
PWY-6629	superpathway of L-tryptophan biosynthesis	1.174	0.537	0.029	0.381
PWY-7446	sulfoglycolysis	1.215	0.525	0.021	0.369
PWY-5028	L-histidine degradation II	1.451	0.707	0.040	0.439
PWY-6749	CMP-legionaminate biosynthesis I	1.606	0.582	0.006	0.138
CODH-PWY	reductive acetyl coenzyme A pathway	1.901	0.389	1.005×10^{-6}	7.721×10^{-5}

PWY-6876	isopropanol biosynthesis	2.621	0.675	0.000	0.006

LogFC: log2 (fold change); Adj *p* values: *p* values corrected by FDR.

Table S4 Differential lipids between vehicle-treated HFD-fed mice and IH-treated HFD-fed mice.

Lipid	Mean of vehicle-treated NCD mice	Mean of vehicle-treated HFD mice	Mean of IH (500 mg/kg)-treated HFD mice	vehicle-treated NCD mice versus vehicle-treated HFD mice		IH (500 mg/kg)-treated HFD mice versus vehicle-treated HFD mice	
				VIP	*p* value	VIP	*p* value
Up-regulated lipids after IH administration (Number: 26)							
LPE(18:2)	9777439.987	2182957.267	2808128.041	1.624	9.369×10^{-6}	1.308	0.024
MePC(31:0e)	6811757.928	1743488.663	2821071.766	1.327	7.766×10^{-6}	1.812	0.001
MePC(33:2e)	17794754.080	13765281.220	17526485.040	1.048	0.066	3.048	0.064
MePC(33:4)	5923938.194	932588.363	1369758.707	1.311	0.000	1.146	0.003
MePC(34:2)	27537527.900	18303392.290	21016579.710	1.579	0.070	2.605	0.058
MePC(34:3)	7578395.585	3991486.753	4698675.750	1.102	0.001	1.364	0.038
MePC(35:4e)	14509749.500	5542578.557	6673392.441	1.760	9.784×10^{-5}	1.712	0.043
MePC(36:3)	10443829.380	3761102.732	4709703.549	1.507	0.001	1.574	0.040
PC(15:0_18:2)	17523161.070	1994829.661	3185871.484	2.307	0.000	1.872	0.006
PC(18:2_18:2)	6394304.916	1511356.958	1934877.872	1.303	2.878×10^{-6}	1.073	0.026
PC(34:2e)	18220250.520	6856919.612	9859020.764	1.983	6.545×10^{-5}	2.966	0.007
PC(34:3)	106495169.600	19131025.960	27667430.290	5.487	0.000	4.739	0.038
PC(34:3e)	6815952.497	1733344.392	2800826.014	1.329	7.853×10^{-6}	1.803	0.001
PC(35:2)	27285655.920	8679544.528	10421634.890	2.543	8.094×10^{-6}	2.270	0.005
PC(35:3)	14954569.910	2291772.163	3358071.955	2.085	0.000	1.783	0.004
PC(36:5e)	17853603.200	13994365.890	17556145.670	1.031	0.061	2.982	0.059
PC(37:2)	10308689.500	2493648.678	3944128.174	1.643	9.290×10^{-5}	2.111	0.001
PC(37:6)	7466368.766	3947860.037	4638147.096	1.091	0.001	1.326	0.050
PC(38:7)	6394304.916	1511356.958	1934877.872	1.303	2.878×10^{-6}	1.073	0.026

PC(38:7e)	14510069.000	5545066.099	6759954.541	1.760	9.890×10^{-5}	1.781	0.040
PC(39:6)	10486917.320	3796434.716	4670334.543	1.508	0.001	1.459	0.070
PC(40:5)	20164068.300	14848439.560	17297907.990	1.281	0.018	2.594	0.023
PC(40:7)	181870119.000	152513199.500	167778331.700	3.136	0.002	5.944	0.095
PC(40:7e)	5861040.133	2061802.215	2764006.650	1.148	1.975×10^{-5}	1.411	0.014
PE(18:2e)	9777439.987	2182957.267	2808128.041	1.624	9.369×10^{-6}	1.308	0.024
SM(t40:7)	7007977.685	2602683.432	3130958.205	1.234	7.615×10^{-5}	1.136	0.069
Down-regulated lipids after IH administration (Number: 4)							
LPC(20:3)	62712467.070	82286688.400	53299922.900	2.223	0.103	8.607	0.049
LPC(20:4)	377372788.000	505646308.400	419747829.400	6.285	0.019	13.945	0.106
MePC(37:3)	5024673.494	8219645.159	7496516.749	1.039	0.001	1.342	0.060
SM(d42:6)	298204.856	11817570.370	8648869.879	1.999	2.723×10^{-5}	2.906	0.033

The standard for significant differential lipids is: $p \leqslant 0.05$ and VIP $\geqslant 1$.

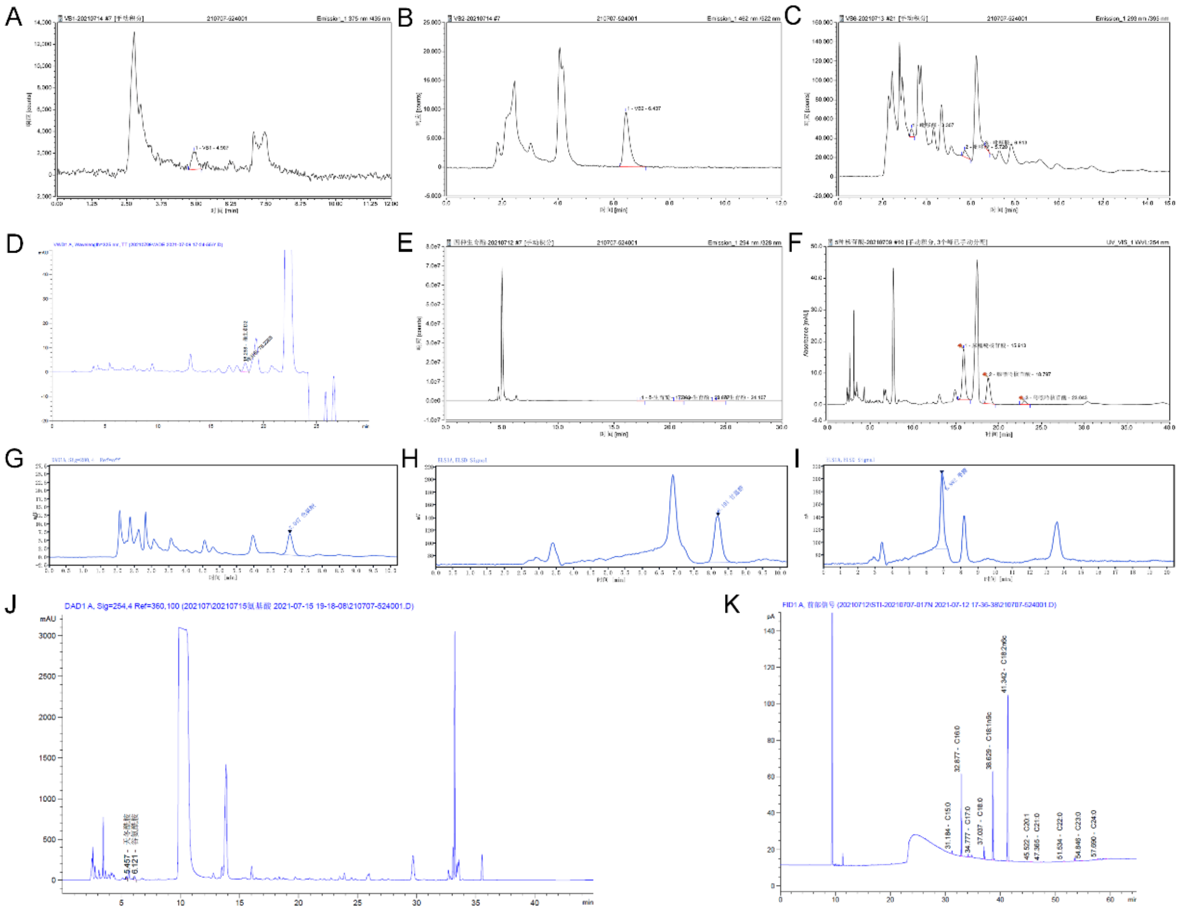

Figure S1 HPLC chromatograms of IH components. (A) Vitamin B1, (B) Vitamin B2, (C) Vitamin B6, (D) Vitamin D2, (E) Vitamin E, (F) Nucleotides, (G) Tryptophan, (H) Mannitol, (I) Fructose, (J) Asparagine/Glutamine and (K) Fatty Acids.

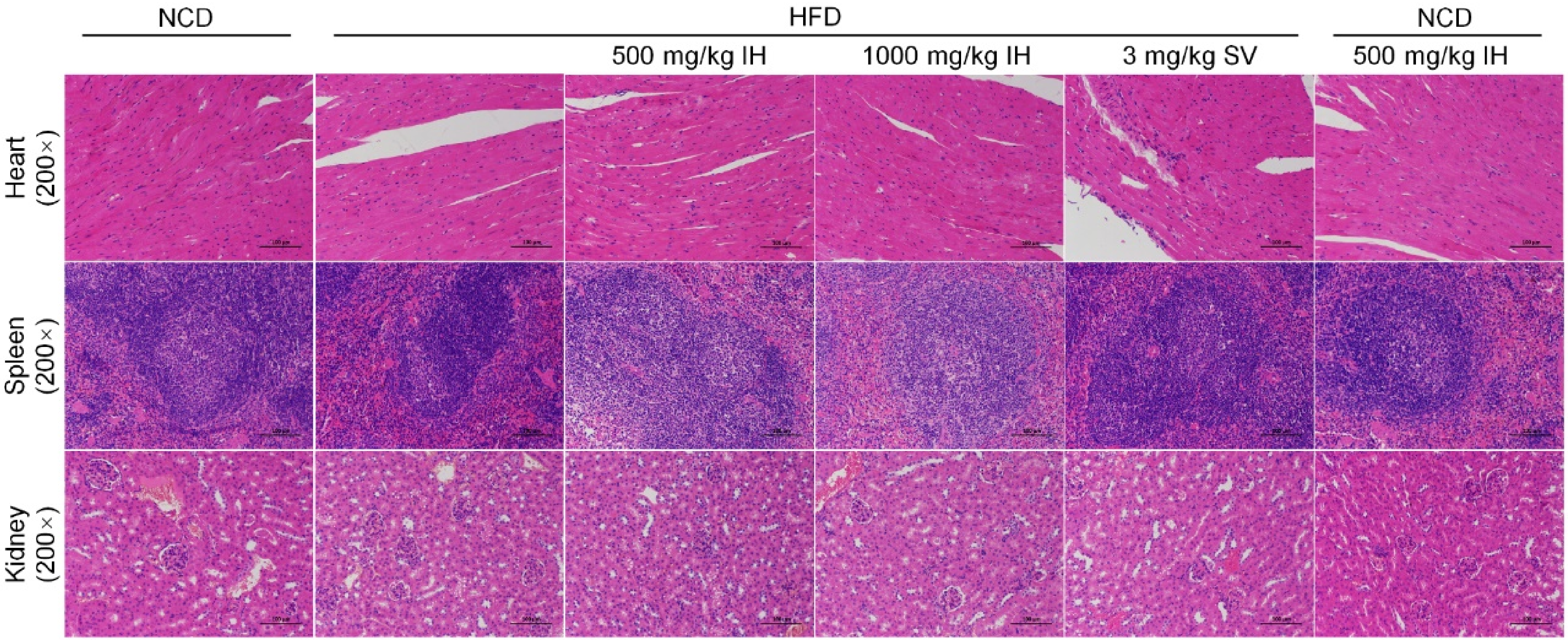

Figure S2 H&E staining of heart, spleen and kidney of mice. IH administration had no effect on the heart, spleen and kidney of mice (200×; scale bar: 100 μm).
